# A genome-wide association study of immune response traits in Canadian Holstein cattle

**DOI:** 10.1186/1471-2164-15-559

**Published:** 2014-07-04

**Authors:** Kathleen A Thompson-Crispi, Mehdi Sargolzaei, Ricardo Ventura, Mohammed Abo-Ismail, Filippo Miglior, Flavio Schenkel, Bonnie A Mallard

**Affiliations:** Department of Pathobiology, Ontario Veterinary College, University of Guelph, 50 Stone Road, Guelph, ON N1G 2W1 Canada; Center for Genetic Improvement of Livestock, University of Guelph, Guelph, ON N1G 2W1 Canada; L’Alliance Boviteq, Saint-Hyacinthe, QC J2T 5H1 Canada; Beef Improvement Opportunities, Guelph, ON N1K 1E5 Canada; Department of Animal and Poultry Science, Damanhour University, Damanhour, Egypt; Canadian Dairy Network, Guelph, ON N1K 1E5 Canada

**Keywords:** Immune response, Dairy cattle, Health, Genome-wide association study, Antibody, Mastitis, Major histocompatability complex, Cytokine

## Abstract

**Background:**

Breeding for enhanced immune response (IR) has been suggested as a tool to improve inherent animal health. Dairy cows with superior antibody-mediated (AMIR) and cell-mediated immune responses (CMIR) have been demonstrated to have a lower occurrence of many diseases including mastitis. Adaptive immune response traits are heritable, and it is, therefore, possible to breed for improved IR, decreasing the occurrence of disease. The objective of this study was to perform genome-wide association studies to determine differences in genetic profiles among Holstein cows classified as High or Low for AMIR and CMIR. From a total of 680 cows with immune response phenotypes, 163 cows for AMIR (81 High and 82 Low) and 140 for CMIR (75 High and 65 Low) were selectively genotyped using the Illumina Bovine SNP50 BeadChip. Results were validated using an unrelated population of 164 Holstein bulls IR phenotyped for AMIR and 146 for CMIR.

**Results:**

A generalized quasi likelihood score method was used to determine single nucleotide polymorphisms (SNP) and chromosomal regions associated with immune response. After applying a 5% chromosomal false discovery rate, 186 SNPs were significantly associated with AMIR. The majority (93%) of significant markers were on chromosome 23, with a similar peak found in the bull population. For CMIR, 21 SNP markers remained significant. Candidate genes within 250,000 base pairs of significant SNPs were identified to determine biological pathways associated with AMIR and CMIR. Various pathways were identified, including the antigen processing and presentation pathway, important in host defense. Candidate genes included those within the bovine Major Histocompatability Complex such as BoLA-DQ, BoLA-DR and the non-classical BoLA-NC1 for AMIR and BoLA-DQ for CMIR, the complement system including C2 and C4 for AMIR and C1q for CMIR, and cytokines including IL-17A, IL17F for AMIR and IL-17RA for CMIR and tumor necrosis factor for both AMIR and CMIR. Additional genes associated with CMIR included galectins 1, 2 and 3, BCL2 and β-defensin.

**Conclusions:**

The significant genetic variation associated with AMIR and CMIR in this study may imply feasibility to include immune response in genomic breeding indices as an approach to improve inherent animal health.

## Background

The inclusion of immune response traits in breeding indices has been suggested to improve inherent disease resistance in dairy cattle [1, 2]. Using a patented test system developed at the University of Guelph, cows with superior cell-mediated (CMIR) and antibody-mediated immune responses (AMIR) can be identified [3]. In one study, Holstein cows classified as having High AMIR were shown to have lower occurrence of mastitis in 2 out of 3 herds tested, improved response to commercial vaccine and increased milk and colostrum quality [4]. High immune response (HIR) cows have also been shown to have decreased incidence of diseases such as mastitis, metritis, ketosis, retained placenta and are less likely to be seropositive for Johne’s disease [5–7]. These previous studies found many benefits of identifying HIR cows in a herd. However, they were performed on one or a few herds in a single region.

Subsequently, immune response profiles were measured on 680 Holsteins from 58 herds across Canada in collaboration with the Canadian Bovine Mastitis Research Network [8]. Significant variation in immune response phenotypes between cows, herds and regions was found, indicating it is possible to classify cows as High, Average or Low Immune Responders on a national scale. Genetic parameters of the immune response traits for these cows were estimated, and AMIR and CMIR were found to be heritable, 0.29 and 0.19, respectively [9]. These heritability estimates are similar to those for production traits [10] demonstrating the feasibility of breeding for enhanced immune response. Cows were classified as High, Average or Low using estimated breeding values (EBV) for AMIR and CMIR. Associations with mastitis were investigated, and High AMIR cows were found to have significantly lower incidence rates of clinical mastitis compared to Average and Low AMIR cows [11]. Also, the Low AMIR cows tended to have the most severe mastitis. These previous studies demonstrate breeding cattle for enhanced immune response, on a national scale, may decrease the incidence and severity of disease in the dairy industry. Further, beneficial associations with some reproductive and longevity traits have been reported, suggesting that breeding for enhanced immune response may also improve longevity and reproductive traits [9].

Genome-wide association studies (GWAS) utilize information on genetic markers or single nucleotide polymorphisms (SNP) evenly spaced across the genome to determine associations with a trait of interest [12]. In cattle, various GWAS have been performed to evaluate genetic differences for a variety of traits like production, reproduction and conformation [13] and for susceptibility or resistance to certain disease such as Johne’s disease [14, 15], bovine tuberculosis [16], mastitis or somatic cell score [17–19]. These studies have been useful for identifying SNP markers and genes associated with a particular disease [20, 21]. However, no GWAS have been performed to evaluate general immune responsiveness in cattle. Therefore, the objective of this study was to use a genome-wide association approach to identify SNP markers, candidate genes and biological pathways associated with AMIR and CMIR. Results of this work are expected to provide insight into the immunological regulation of general antibody and cell-mediated immunity in cattle, as well as demonstrate the potential to include immune response traits in genomic selection indices to decrease the occurrence of disease and improve animal health in the dairy industry.

## Methods

### Animals

Immune responses traits (CMIR and AMIR) of 680 lactating Holsteins, outside the peripartum period, from 58 herds across Canada were evaluated [8] in collaboration with the Canadian Bovine Mastitis Research Network. For validation, a total of 543 Holstein bulls were immune response phenotyped in collaboration with the Semex Alliance. All experimental procedures were approved by the Animal Care Committee of the University of Guelph under guidelines of the Canadian Council of Animal Care.

### Immune response traits

As described and reported previously, cows and bulls were immunized with both a type 1 and a type 2 test antigen to induce CMIR and AMIR, respectively [8]. A delayed-type hypersensitivity test to the type 1 test antigen was used as an indicator of CMIR. AMIR was evaluated by measuring serum antibody of the IgG1 isotype to the type 2 test antigen by enzyme-linked immunosorbent assay (ELISA) on Day 0, 14 and 21 of the immunization protocol. Genetic parameters and breeding values of the adaptive immune response traits AMIR and CMIR in these herds have been estimated and reported previously [9]. The CMIR had an estimated heritability of 0.19 while estimates for AMIR were 0.27 and 0.38 on Day 14 and 21, respectively. For use in the genome-wide association study, cows were ranked on the average AMIR at Day 14 and Day 21 and bulls were ranked on AMIR at Day 14. Cattle with an EBV > + 1 or < - 1 standard deviation from the mean were considered High Immune Responders or Low Immune Responders, respectively.

### Genotyping and quality control

Selective genotyping was used to increase the probability of finding significant markers for these traits despite the relatively small number of individuals that were genotyped in this first study. A total of 163 cows for AMIR (81 High and 82 Low) and 140 cows for CMIR (75 High and 65 Low) were selectively genotyped using the Bovine SNP50 BeadChip (Illumina, San Diego, CA). Hair follicles were collected as a source of DNA. DNA was extracted by Maxxam Analytics (Guelph, Ontario, Canada) and genotyping performed by DNA Landmarks (Saint-Jean-sur-Richelieu, Quebec, Canada). Bull genotypes were provided by the Semex Alliance. For AMIR there were 83 High and 81 Low responders and for CMIR 74 High and 72 Low responders. On average, there were 4.78 missing genotypes for cows and 2.38 for bulls, which were imputed using FImpute [22]. A total of 45,187 SNP markers were considered for the association analysis based on USDA quality control measures [23]. Additional quality control measures applied to the data included the exclusion of SNP markers with a minor allele frequency (MAF) of less than 0.05 and individuals with a call rate equal or less than 0.85.

### Statistical analysis

A generalized quasi-likelihood score method [24] was used to determine SNP markers significantly associated with AMIR and CMIR for cows and bulls separately. This method accounts for the background polygene effect by using pedigree-based relationships among animals and is not biased by selective genotyping, since it is based on a logistic regression approach. Pedigrees were obtained from the Canadian Dairy Network and included 29,402 and 19,189 for the cows’ and bulls’ pedigree, respectively. In order to account for multiple comparisons, a chromosomal False Discovery Rate (FDR) of 0.05 was applied [25].

### Candidate gene discovery and pathway analysis

Significant SNPs were mapped to corresponding or nearby genes using NGS-SNP [26]. Genes 250,000 base pairs (bp) up or downstream of the significant SNP were obtained for pathway analysis. The identified genes were submitted to database for annotation and visualization and the integrated discovery (DAVID) bioinformatics resource 6.7 to perform enrichment analysis in order to determine biological pathways associated with AMIR and CMIR [27, 28].

## Results

**Figure 1:**
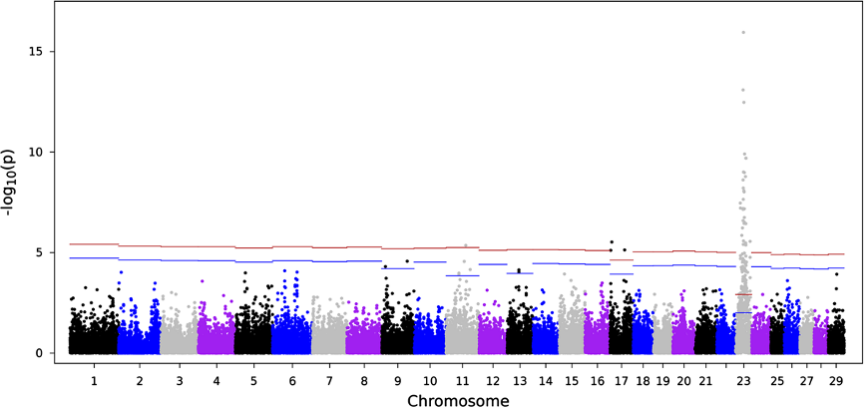
**Manhattan plot for antibody-mediated immune response (AMIR) in Holstein cows.** The x-axis is the position of each SNP on the bovine chromosomes and the y-axis is the –log_10_ *P*. The red and blue lines indicate chromosome-wise 5% and 1% false discovery rate, respectively.

**Figure 2:**
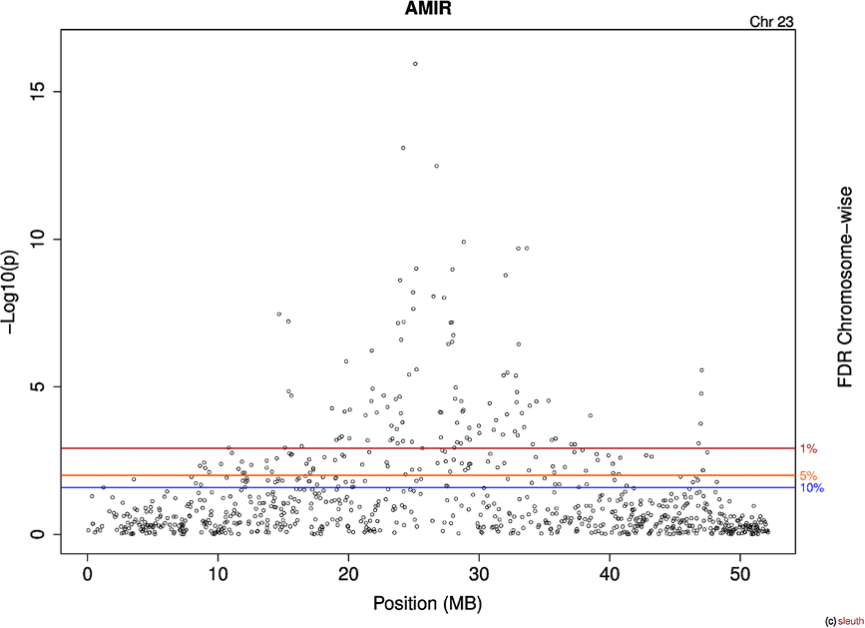
**Manhattan plot for antibody-mediated immune response (AMIR) SNPs on chromosome 23 of Holstein cows.** The x-axis is the position of each SNP on the bovine chromosome and the y-axis is the –log_10_ *P*. The lines indicate chromosome-wise false discovery rate (FDR).

The dataset contained 40,935 SNPs for AMIR and 40,973 SNPs for CMIR after applying quality control measures. For AMIR 18 (8 High and 10 Low) individuals were removed for low call rates (<0.85). For CMIR, 7 individuals were removed from the analysis for the same reason (3 High and 4 Low). Figure 1 shows the Manhattan plot of the –log10(*p*) for all markers for AMIR. A total of 2,741 SNPs were significantly (comparison-wise *P* < 0.05) associated with AMIR. After accounting for multiple comparisons, 186 SNPs remained significant at a chromosome-wise 5% FDR. The majority (93%) of the SNP markers associated with AMIR were on chromosome 23 (173/186) and this chromosome lost the fewest SNPs after accounting for multiple comparisons. The smallest p values were observed for chromosome 23 and therefore this chromosome lost only 32% of the significant SNPs after applying the FDR, versus the other chromosomes that lost > 90%. Figure 2 shows the Manhattan plot results for AMIR for chromosome 23 only.

**Figure 3:**
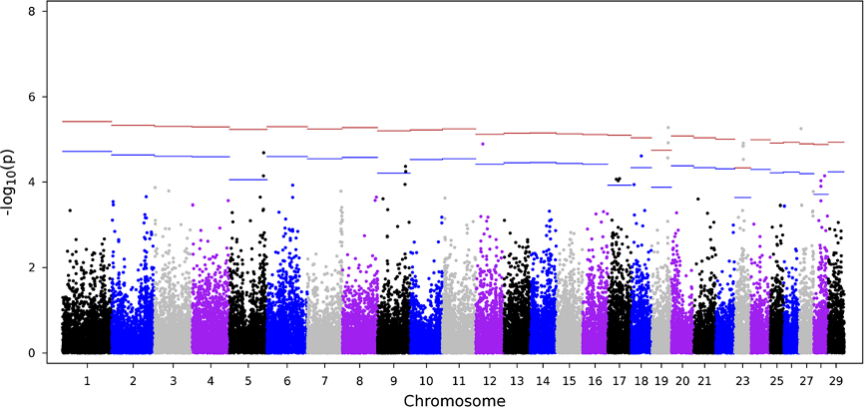
**Manhattan plot for the cell-mediated immune response (CMIR) in Holstein cows.** The x-axis is the position of each SNP on the bovine chromosomes and the y-axis is the –log_10_ *P*. The red and blue lines indicate chromosome-wise 5% and 1% false discovery rate, respectively.

**Figure 4:**
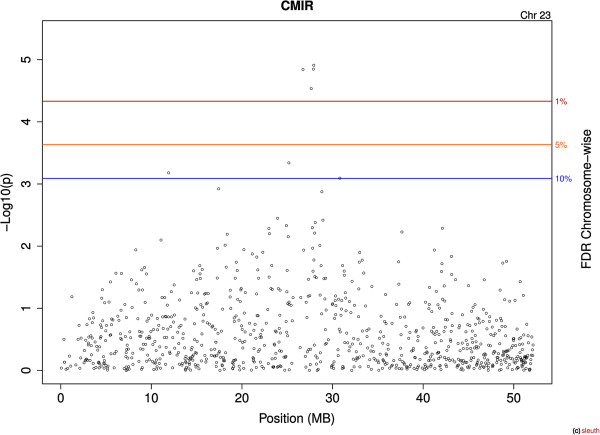
**Manhattan plot for the cell-mediated immune response (CMIR) SNPs on chromosome 23 of Holstein cattle.** The x-axis is the position of each SNP on the bovine chromosome and the y-axis is the –log_10_ *P*. The lines indicate chromosome-wise false discovery rate (FDR).

Figure 3 shows the Manhattan plot of the –log10(*p*) for all markers for CMIR. A total of 2,976 markers were significantly (comparison-wise *P* < 0.05) associated with CMIR, and 21 remained significant after accounting for multiple comparisons. Chromosome 23 contained the largest proportion of significant markers (4/21), and all 4 markers were also significant for AMIR. Figure 4 shows the Manhattan plot results for CMIR for chromosome 23 only.

For AMIR, 428 genes were found within 250,000 bp of the 186 SNP markers significant at FDR < 0.05. Table 1 shows the top 10 significant SNPs associated with AMIR and the genes within 250,000 base pairs of the markers. Candidate genes included those within the bovine Major Histocompatability Complex such as BoLA-DQ, BoLA-DR and the non-classical BoLA-NC1, the complement system including C2 and C4, and cytokines including IL-17A, IL17F and tumor necrosis factor. All genes were submitted to DAVID and 67 genes were enriched to 12 biological pathways (*P* < 0.05) through Kyoto Encyclopedia of Genes and Genomes (KEGG) (Table 2). The antigen processing and presentation pathway had 11 genes significantly associated with this pathway, mainly due to the BoLA genes found on chromosome 23.

**Table 1:** **Top ten single nucleotide polymorphism (SNP) markers significantly (chromosome-wise false discovery rate < 0.05) associated with antibody-mediated immune response in Holstein cows and genes within 250,000 base pairs flanking the markers**

BTA	SNP	Location (bp)	-log _10_ *P*	Entrez gene ID
23	ARS-BFGL-NGS-111879	25,109,188	15.95	BolA-DQA1, ELOVL5, FBXO9, GCM1, GSTA3, GSTA4, GSTA5, ICK
23	Hapmap50029-BTA-55899	24,181,053	13.10	IL17A, IL17F, MIR133B, MIR206
23	BTA-27247-no-rs	26,736,263	12.49	BTN3A2, NOTCH4, TSBP
23	ARS-BFGL-NGS-105563	28,819,118	9.91	GABBR1, MOG, PPP1R11, TRIM10, TRIM15, TRIM26, TRIM31, TRIM40, UBD, ZNRD1
23	ARS-BFGL-BAC-3611	33,645,739	9.70	-
23	Hapmap57845-rs29014813	32,998,188	9.69	ACOT13, ALDH5A1, FAM65B, GMNN, GPLD1, MRS2, TDP2
23	Hapmap44002-BTA-110636	25,178,791	9.01	BOLA-DQA1, BOLA-DQB, ELOVL5, FBXO9, GCM1, GSTA3, GSTA4, ICK
23	BTA-55821-no-rs	27,944,066	8.98	ATAT1, BOLA, CCHCR1, CDSN, DDR1, DHX16, FLOT1, GTF2H4, IER3, KIAA1949, MDC1, MICB, MRPS18B, NRM, POU5F1, PPP1R10, PSORS1C2, SFTA2, TCF19, TUBB
23	ARS-BFGL-NGS-37272	32,025,158	8.78	HIST1H2BA, LRRC16A, SCGN, SLC17A1, SLC17A3, SLC17A4
23	Hapmap41584-BTA-56031	24,939,249	8.20	ELOVL5, FBXO9, GCM1, GSTA1, GSTA3, GSTA3, GSTA4, GSTA5, ICK, TMEM14A, TRAM2

**Table 2:** **Biological pathways significantly (** *P* **< 0.05) associated with antibody mediated immune response of Holstein cattle**

KEGG ^1^ pathway	Count	%	*P*value	Benjamini ^2^
Systemic lupus erythematosus	19	4.7	2.1E-10	2.2E-08
Type I diabetes mellitus	9	2.2	3.3E-06	1.7E-04
Antigen processing and presentation	11	2.7	4.1E-06	1.4E-04
Olfactory transduction	32	7.9	4.5E-06	1.2E-04
Graft-versus-host disease	8	2.0	5.7E-06	1.2E-04
Allograft rejection	8	2.0	1.4E-05	2.4E-04
Asthma	6	1.5	2.4E-04	3.5 E-03
Autoimmune thyroid disease	7	1.7	5.5 E-04	7.2 E-03
Viral myocarditis	7	1.7	3.5 E-03	4.0 E-02
Intestinal immune network for IgA production	5	1.2	2.7 E-02	2.5 E-02
Spliceosome	8	2.0	2.9 E-02	2.4 E-02

^1^Kyoto Encyclopedia of Genes and Genomes.

^2^Benjamini = False Discovery Rate α = 0.1.

For CMIR, 98 genes were within 250,000 bp of the 17 significant markers. Table 3 shows the top 10 significant markers and genes associated with CMIR. Genes related to immune response included BoLA-DQ, C1q associated with the complement system and the cytokine receptor IL-17RA as well as tumor necrosis factor. Additional genes associated with CMIR included galectins 1, 2 and 3, BCL2 and β-defensin. The CMIR genes were not significantly (*P* < 0.05) enriched in pathways using DAVID, but 3 genes were enriched in the natural killer cell mediated cytotoxicity pathway (*P* = 0.061).

**Table 3:** **Top ten single nucleotide polymorphism (SNP) markers significantly (chromosome-wise false discovery rate < 0.05) associated with the cell-mediated immune response in Holstein cows and genes within 250,000 base pairs flanking the markers**

BTA	SNP	Location (bp)	-log _10_ *P*	Entrez gene ID
19	ARS-BFGL-NGS-101995	53,606,174	5.28	RBFOX3, CBX8, CBX2
27	ARS-BFGL-NGS-38750	4,845,275	5.25	ZNF705A, AGPAT5, SPAG11B, TAP, DEFB103B
19	UA-IFASA-7781	53,963,109	4.92	CANT1, LGALS3BP, TIMP2, C1QTNF1, RBFOX3, USP36
23	Hapmap46836-BTA-55820	27,923,154	4.91	TCF19, SFTA2, MICB, NRM, FLOT1, PSORS1C2, CDSN, POU5F1, MDC1, CCHCR1, BOLA, DDR1, GTF2H4, TUBB, DHX16, ATAT1, MRPS18B, KIAA1949, IER3
12	BTA-86812-no-rs	23,174,195	4.89	LHFP, STOML3, PROSER1, NHLRC3
23	ARS-BFGL-NGS-16619	27,887,914	4.85	POU5F1, PSORS1C2, IFITM3, TCF19, MICB, BOLA, IER3, FLOT1, TUBB, CCHCR1, NRM, DDR1, CDSN, GTF2H4, MDC1, KIAA1949, SFTA2
23	BTA-27247-no-rs	26,736,263	4.84	NOTCH4, BTN3A2, TSBP
5	ARS-BFGL-NGS-627	109,734,406	4.69	BID, LGALS2, CDC42EP1, GGA1, MICAL3, BCL2L13, PEX26, TUBA8, ATP6V1E1
5	ARS-BFGL-NGS-10118	109,774,563	4.69	TUBA8, MICAL3, BID, BCL2L13, CDC42EP1, LGALS2, GGA1, PDXP, LGALS1, NOL12, MIR2438, ATP6V1E1, PEX26
18	BTA-24218-no-rs	32,925,593	4.61	CDH11

**Figure 5:**
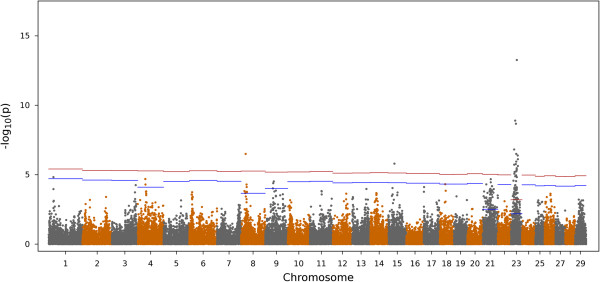
**Manhattan plot for the antibody-mediated immune response in Holstein bulls.** The x-axis is the position of each SNP on the bovine chromosomes and the y-axis is the –log_10_ *P*. The red and blue lines indicate chromosome-wise 5% and 1% false discovery rate, respectively.

**Figure 6:**
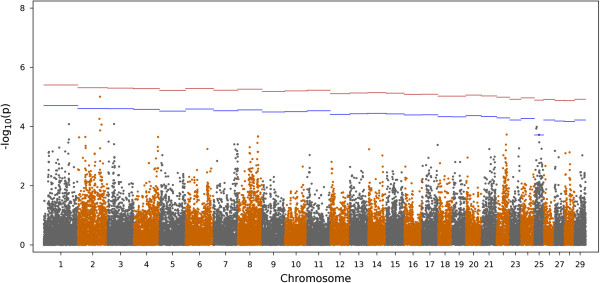
**Manhattan plot for the cell-mediated immune response in Holstein bulls.** The x-axis is the position of each SNP on the bovine chromosomes and the y-axis is the –log_10_ *P*. The red and blue lines indicate chromosome-wise 5% and 1% false discovery rate, respectively.

Results for AMIR and CMIR were validated in a population of bulls that have been immune response phenotyped with the same protocol as that used for cows. No bulls were removed from the analysis due to low individual call rates. For AMIR, the peak on chromosome 23 was confirmed (Figure 5). The number of significant SNP markers in bulls for both AMIR (Figure 5) and CMIR (Figure 6) confirms the large degree of genetic variation in these immune response traits. The antigen processing and presentation pathway was significantly (*P* < 0.0001) associated with AMIR in the bulls, verifying results found in cows.

## Discussion

This study was the first genome-wide association study for general adaptive immune responsiveness in dairy cattle. Previous GWAS have evaluated differences in resistance or susceptibility to certain diseases; however, the approach proposed here may identify SNP profiles associated with general disease resistance, since cows with superior immune responses are known to have a lower occurrence of disease [7, 11]. The current study found significant variation in SNP profiles between cows classified as High or Low for AMIR and CMIR, indicating that it may, one day, be possible to identify animals with superior immune responses and therefore disease resistance based on genetic profiles. The significant variation in SNP profiles of cows for both AMIR and CMIR were confirmed in an independent population of Holstein bulls, providing strength to the results found here. The majority of significant SNPs were found on chromosome 23 for AMIR, with the antigen processing and presentation pathway being significantly associated with AMIR. Again, the significant peak of markers found on chromosome 23 in cows and the antigen processing and presentation pathway were confirmed in the bulls, which were not closely related to the population of tested cows.

In cattle, the major histocompatibility complex (MHC), known as bovine leukocyte antigen (BoLA), is located on chromosome 23 and is well known as a location of major genes associated with immune response and disease resistance [29, 30]. The MHC is involved in processing and presenting host and pathogen peptides to the cells of the immune system. The MHC class I is involved in processing and presenting endogenous peptides to CD8+ T cells [31], whereas MHC class II tends to present exogenous or extracellular peptides to CD4+ T cells [32, 33], which then mediate appropriate host responses. Given that the majority of SNPs associated with AMIR in this study were located on chromosome 23 and the function of this gene region is to mediate effective adaptive immune responses, it is expected that the antigen and processing pathway was significantly associated with AMIR. The antigen processing and presentation pathway was also significant in the independent bull population, validating the results found in cows. For CMIR, although the enriched genes list in this pathway was not significant, a candidate gene was identified in BolA.

Bovine MHC I is under control of 6 loci, with combinations of two or three haplotypes expressed allowing for high degree polymorphism and diversity in this region [34]. On the other hand, the class II region consists of genes encoding two proteins, DR and DQ, with DRA being monomorphic and DRB and DQ regions being highly polymorphic. The DQ locus is duplicated, and therefore many haplotype combinations allow the class II region to maintain a high degree of polymorphism [35]. As suggested recently, it is possible that deliberate selection for production in dairy cattle has decreased the diversity in the MHC region explaining the associated increase in diseases like mastitis with known links to BoLA [36]. Selection strategies to maintain diversity and increase heterozygosity in MHC genes may be important in order to breed robust cattle capable of responding appropriately to a variety of challenges including both intra- and extracellular pathogens. The high degree of variation on chromosome 23 associated with AMIR in the current study, in particular within BolA, suggests that selection for immune responsiveness might be an approach to maintain diversity in MHC genes.

The relationship between BoLA class II and resistance or susceptibility to mastitis has been known for over 20 years [37–40]. T cell proliferative responses have also been demonstrated to be dependent on the bovine MHC II [41]. The high antibody responding cows used in this study have previously been demonstrated to have a lower incidence rate of clinical mastitis compared to the low antibody responding counterparts that were selected for selective genotyping [11]. Therefore, an association with this highly polymorphic and complex gene region could be expected. Associations between the immune response traits AMIR and CMIR and BoLA have been demonstrated previously [38, 42]. Rupp et al. [42] found different allele combinations associated with AMIR and CMIR, and suggested they were independent or negatively correlated genetically, which has been shown previously [9]. Additionally, certain BolA allele combinations were associated with increased risk of mastitis.

In this study, interleukin 17 (IL-17) was a candidate gene associated with AMIR and its receptor, IL17RA, associated with CMIR. IL-17 is a proinflammatory cytokine produced mainly by CD4+ T cells of the Th17 lineage, but also dendritic cells [43, 44]. IL-17 regulates innate host defenses by stimulating cells such as fibroblasts and epithelial cells to produce IL-6, IL-8 and granulocyte colony stimulating factor (G-CSF) which in turn recruit neutrophils contributing to the development of acute inflammation [44]. IL-17A and IL-17 F have been implicated in modulation of mammary gland immune responses to mastitis causing bacteria [45]. An *in vitro* challenge model using a bovine mammary epithelial cell culture with components of *Staphylococcus aureus*, a common mastitis-pathogen, found an increase in gene expression of proteins with antibacterial properties in the presence of IL-17A and IL-17 F, and expression was increased in the presence of TNFα [45], another candidate gene associated with AMIR and CMIR in the present study. Milk somatic cells isolated from cows identified as positive for *Staphlyococcus aureus* mastitis have been found to have an increased expression of IL-17 compared to blood mononuclear cells [46]. IL-17 has also been implicated in immune responses to *Neospora caninum* [47], as well as *Mycobacterium tuberculosis* vaccination [48]. Given that the antibody and cell-mediated immune response traits evaluated in the current study represents an overall ability for the cow to make a robust antibody and cellular responses, it is logical that cytokines like IL-17 and TNFα, which are involved in host defence, were associated with these traits.

Candidate genes associated with CMIR included galectins which have been shown to be induced in response to gastrointestinal nematode infection in cattle [49] and sheep [50], but are better known for their role in reproduction [51, 52]. CMIR was also associated with the β-defensins which have a variety of roles in protection from pathogens and regulation of immune responses and reproduction [53]. More specifically, CMIR was associated with tracheal associated protein (TAP), a β-defensin which has been found to be important in killing bacteria that can cause pneumonia in cattle [54]. Candidate genes associated with the classical pathway of the complement system were also found to be associated with both AMIR and CMIR. The complement system is an important component of the innate host defense, critical in initiating adaptive responses such as those measured in the current study. The variety of immune response related genes identified here provide an opportunity for future studies on the genetic regulation of these molecules in general adaptive immune responses.

In the dairy industry, selection for complex traits, such as production, has been successful. In cattle, the vast majority of complex traits are under polygenic control, having many genes with small effects contributing to the variation of each trait, with some exceptions such as the *diacylglcerol O-acyltransferase* (DGAT1) [55] and ATP binding cassette subfamily G member 2 (ABCG2) [56] associated with milk production, myostatin associated with double muscle in beef cattle [57] and MHC DQ haplotypes associated with mastitis [39]. For traits such as general immune response where it is impossible for a single gene to regulate the trait, using estimated breeding values or genomic estimated breeding values may overcome the issue of complex regulation by selecting for all genes controlling the trait, without necessarily knowing the genes themselves [58].

Genomic selection makes use of genomic estimated breeding values (GEBV) that are estimated based on the sum of marker effects evenly spaced across the genome [59]. Once a substantial reference population with accurate phenotypes and genotypes has been established, it is possible to estimate GEBV for animals without phenotypes. The advent of genomic selection has significantly increased the rate of genetic gain in dairy cattle mainly by reducing the generation interval [60], and could make it feasible to include new phenotypes such as methane production or health in breeding indices [61]. However, the inclusion of new traits in genomic breeding indices will require a substantial reference population with accurate phenotypes and genotypes. This was the first GWAS for general antibody-mediated and cell-mediated immune responses in dairy cattle. Despite the limited number of genotyped animals in this study, several significant markers, candidate genes and pathways were identified. A study based on a larger number of animals with both genotypes and phenotypes would be useful in order to confirm these initial results. However, significant variation found here suggests it may be possible to calculate GEBV for immune response in the future. Moreover, in the future, a larger dataset and the use of sequencing and/or imputation to increase the density of markers may also help identify informative markers within novel genes for AMIR and CMIR.

## Conclusion

This study found a number of significant SNP markers associated with High and Low general antibody and cell-mediated immune responses of Holstein cattle, suggesting it may be possible to calculate genomic breeding values for these traits and include them in breeding indices to decrease the incidence and severity of disease in the dairy industry. Results were validated in a population of Holstein bulls not closely related to the cows. The immune system provides the main defense against pathogenic micro-organisms and as such has the ability to vary the response in accordance with the nature of the invading pathogen or immunizing agent. This system is therefore under complex genetic regulation and individuals differ in their immune response profiles with protective responses not necessarily identical between individuals. The immune system is also dynamic in its capacity to deal with the variation found within and across various pathogens. This was the first genome-wide association study for general antibody and cell-mediated immune responses in cattle. Despite the relatively small number of genotyped individuals, it provides encouraging evidence in support of future studies based on a larger reference population that could lead to the estimation of for genomic breeding values for immune response.
